# Lower Temperature Cultures Enlarge the Effects of *Vitreoscilla* Hemoglobin Expression on Recombinant *Pichia pastoris*

**DOI:** 10.3390/ijms131013212

**Published:** 2012-10-15

**Authors:** Jyh-Ming Wu, Shin-Yao Wang, Wei-Chang Fu

**Affiliations:** 1 Department of Chemical and Materials Engineering, Chinese Culture University, 55, Hwa-Kang Rd., Yang-Ming-Shan, Taipei 111-14, Taiwan; E-Mail: fu_95244611@yahoo.com.tw; 2 Institute of Nanomaterials, Chinese Culture University, 55, Hwa-Kang Rd., Yang-Ming-Shan, Taipei 111-14, Taiwan; E-Mail: alioth77213@hotmail.com

**Keywords:** *Vitreoscilla* hemoglobin, lower temperature, *Pichia pastoris*, AOX 1 promoter, recombinant protein production

## Abstract

An heterologous expression of *Vitreoscilla* hemoglobin (VHb) for improving cell growth and recombinant protein production has been successfully demonstrated in various hosts, including *Pichia pastoris*. Lower temperature cultures can enhance target protein production in some studies of *P. pastoris*. In this study, the strategy of combining heterologous VHb expression and lower temperature cultures in *P. pastoris* showed that final cell density and viability of VHb^+^ strain at 23 °C were higher than that at 30 °C. In addition, the effects of VHb expression on recombinant β-galactosidase production and oxygen uptake rate were also higher at 23 °C than at 30 °C. Consequently, lower temperature cultures can enlarge VHb effectiveness on cell performance of *P. pastoris*. This is because VHb activity obtained at 23 °C cultures was twofold higher than that at 30 °C cultures, due to a different heme production. This strategy makes *P. pastoris* an excellent expression host particularly suitable for increasing the yields of the low-stability and aggregation-prone recombinant proteins.

## 1. Introduction

*Vitreoscilla* hemoglobin (VHb), which is synthesized by the strict aerobic bacterium *Vitreoscilla*, aids the survival and growth of this organism in oxygen-poor environments. The putative function of VHb is to trap oxygen at low extracellular concentrations and deliver it to terminal respiratory oxidases that enhance respiratory efficiency and ATP synthesis [1]. Metabolic engineering studies have demonstrated that intracellular expression of VHb in different hosts, including prokaryotes and eukaryotes, leads to enhanced oxygen availability, increased cell growth, and improved metabolite and recombinant protein production [2,3].

The methylotrophic yeast *Pichia pastoris* has become a promising host for producing large quantities of heterologous proteins because of the availability of a strong, tightly regulated, methanol-inducible alcohol oxidase (AOX1) promoter. *P. pastoris* has the potential for high expression levels, efficient extracellular protein secretion, proper protein folding, post-translational modifications such as glycosylation and disulfide bond formation and high cell density growth on a minimal medium [4,5].

VHb is a suitable oxygen-uptake improving protein for expression in *P. pastoris* because of its high oxygen trapping and releasing ability, enabling it to satisfy extremely high oxygen demand during fermentations. In our previous study [6], intracellular expression of VHb can improve cell performance and recombinant β-galactosidase production in *P. pastoris* VHb^+^ strain cultured at 30 °C, under different aeration conditions.

Recently, some studies have reported that lower temperature cultures at 20–25 °C in *P. pastoris* can improve the target protein production, lower the cell lysis, reduce the release of intracellular proteases to culture medium and decrease the proteolytic activity [7–9]. Therefore, the combinational strategy of VHb technology and lower temperature technique is worthy for the study of the synergetic effect on cell performance of *P. pastoris* and to determine if VHb beneficial effect can be enlarged by lower temperature cultures. Based on our results, the proposed strategy is practical and effective.

## 2. Results and Discussion

### 2.1. Construction of Recombinant Strains

A recombinant *P. pastoris* strain capable of cytosolic co-expression of VHb and β-galactosidase under the control of AOX1 promoter was constructed in our previous study [6]. Briefly, the *vgb*-bearing pP-SVC plasmid was introduced into *P. pastoris* strain GS115/Z_A_ to generate the GS115/Z_A_SVC strain (VHb^+^ strain), whereas the control strain VHb^−^ was constructed by transforming the empty vector pPICZA into the GS115/Z_A_ strain. VHb expression was confirmed by Western-blotting analysis and biological activity was verified by CO-difference spectra assay. The results showed that VHb was functionally expressed in the cytoplasm by VHb^+^ strain [6].

### 2.2. Effect of VHb Expression on Biomass at Lower Temperatures

**Figure 1:**
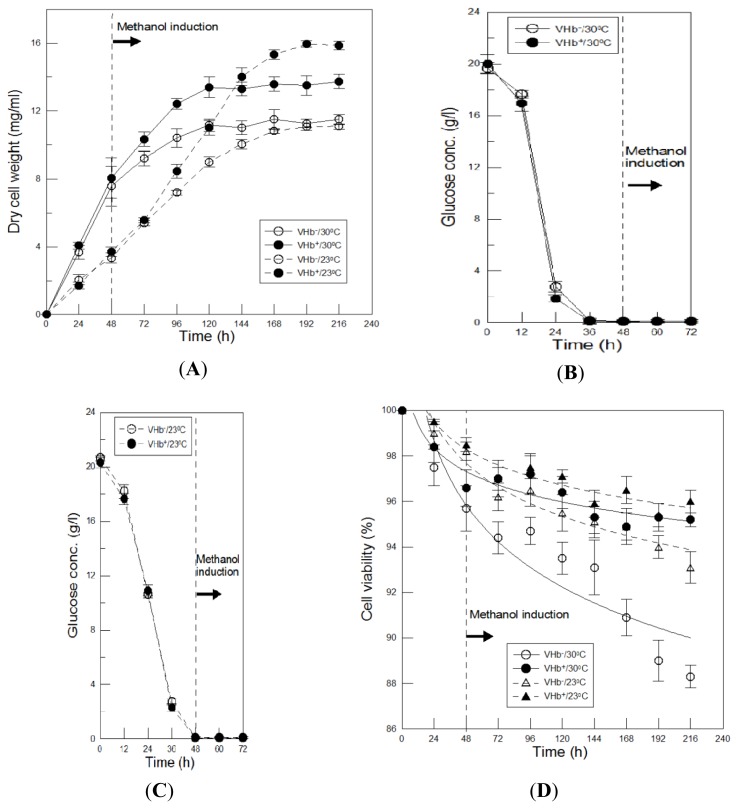
Time courses of cell growth (**A**), glucose concentration (**B**,**C**) and cell viability (**D**) of *Vitreoscilla* hemoglobin (VHb)^+^ and VHb^−^ strains cultured at two temperatures. Data are the means of three independent replicates. Error bars indicate standard deviations.

Cell growth of VHb^+^ and VHb^−^ strains incubated at 250 rpm in shake-flasks under two temperatures of 30 °C and 23 °C were compared to study the effects of VHb expression and lower temperature cultures on *P. pastoris* biomass. As shown in Figure 1A, the two strains growth during the first 48 h of 30 °C cultures were almost identical. After glucose as well as ethanol completely depleted (Figure 1B) and then methanol added at 48 h, the differences in cell growth occurred immediately and increased with culture time caused by induced VHb expression. The maximal biomass of VHb^+^ and VHb^−^ strains reached 13.7 and 11.4 mg-DCW/mL respectively, which was enhanced 20% by the effect of VHb expression. Both strains also displayed identical cell growth during the first 72 h of 23 °C cultures, thereafter, their growth differences significantly increased with time. VHb^+^ and VHb^−^ strains can grow to maximal values of 16.0 and 11.2 mg-DCW/mL respectively, which was a substantial increase of 43% by VHb effect. Therefore, VHb effect on biomass was positive at both temperatures, however, the effect was more pronounced at 23 °C.

Since lower temperature cultures are not favorable for *P. pastoris* growth, the biomass of two strains during the first 72 h of the 23 °C cultures were only half of those at 30 °C. The glucose consumption rates of two strains at 23 °C were also slower than that at 30 °C (Figure 1B,C). However, VHb^−^ strain cultured at two temperatures can reach the same final cell density with different kinetics. At 30 °C most of biomass was produced before methanol induction and growth ceased after induction, while at 23 °C growth continued for a prolonged time. For VHb^+^ strain, growth was faster after induction than for the VHb^−^ strain at 30 °C as well as at 23 °C, leading to more biomass produced. After 144 h of 23 °C cultures cell density of VHb^+^ strain surpassed itself at 30 °C, and at the end of cultures biomass was increased by 17%. Thus, VHb expression in *P. pastoris* at lower temperatures can further enhance final cell density. Additionally, growth phase was prolonged significantly by VHb expression, resulting in enhanced biomass accumulation. This implies that substrates of glucose and methanol were used with better efficiency as to increasing the yield coefficient *Yx*/*s*, since no remaining substrates concentrations were detected at the end of cultures.

The viabilities of VHb^+^ and VHb^−^ strains at both temperatures were also compared. As shown in Figure 1D, the differences in the viabilities of two strains increased with culture time at both temperatures. The decreasing rates of both strains were slower at 23 °C and eventually obtained higher viabilities than that at 30 °C. At 23 °C the viabilities of VHb^+^ and VHb^−^ strains at the end of 216 h of cultures were 96.0% and 93.1% respectively, whereas at 30 °C they were 95.2% and 88.4% respectively. Evidently, VHb expression can benefit the viability at both temperatures. Additionally, the effect of lower temperature cultures on cell viability was higher in VHb^−^ strain than in VHb^+^ strain. This is due to the viability of VHb^+^ strain was already over 95%, thus a further increase was more difficult. Lower temperature cultures can reduce cell death as to increase cell viability in this study. The phenomenon is also observed in other *P. pastoris* studies [10,11], probably because AOX1 activity is higher at lower temperatures so that the toxicity of methanol to cells can be reduced [8].

### 2.3. Analysis of VHb Protein at Two Temperatures

**Figure 2:**
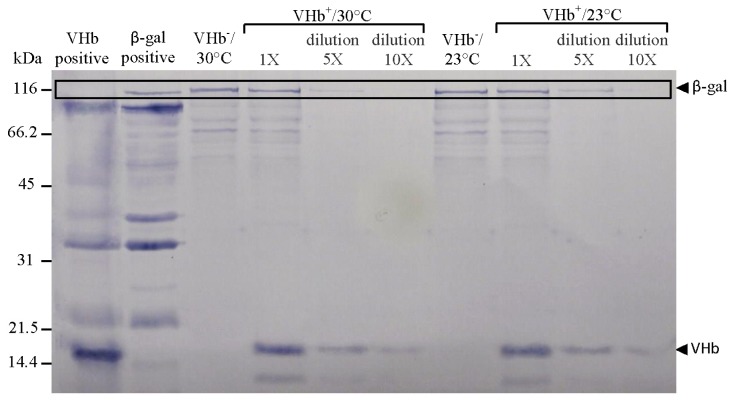
Western-blotting analysis of proteins expressed by VHb^+^ and VHb^−^ strains at the end of 216 h of culture under two temperatures.

It proved worthwhile to investigate why VHb effect on biomass was higher at 23 °C than at 30 °C. First, VHb expression levels were analyzed. Cells were harvested at the end of 216 h of cultures and adjusted to the same OD_600_ before being disrupted. Western-blotting technique was then used to confirm the expressed protein and evaluate protein expression levels. Unexpectedly, the levels of expressed VHb polypeptides (globin portion) at two temperatures were nearly equal (Figure 2). This finding differed from what we first thought, namely, 23 °C cultures could enhance VHb expression (primarily based on the studies reporting that lower temperature cultures can improve recombinant protein expression in *P. pastoris* [10–12]) as to increase VHb effect on biomass. Evidently, VHb expression cannot be increased by lower temperature cultures.

**Figure 3:**
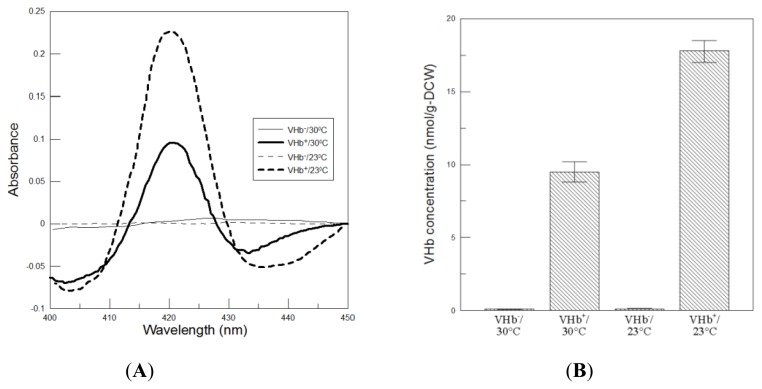

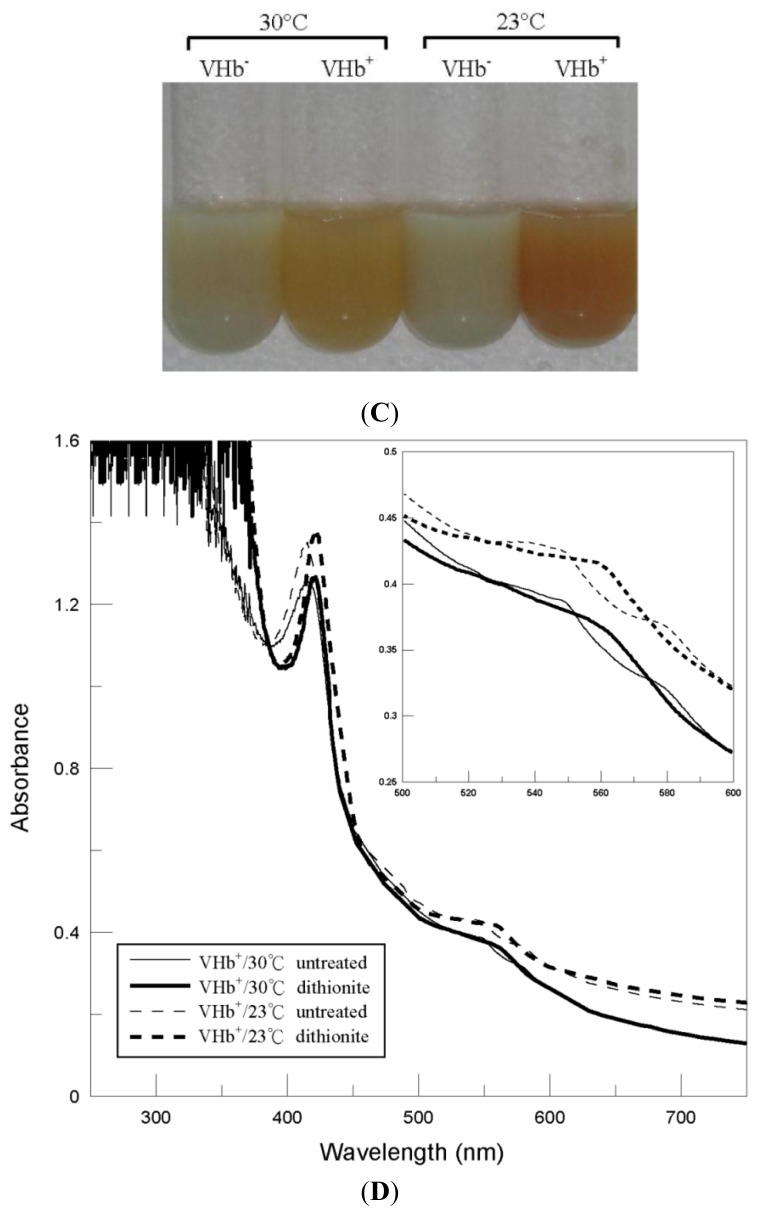
Analysis of VHb expression at the end of 216 h of culture at two temperatures. (**A**) CO-difference spectra of crude protein extracts. The difference in absorbance between CO-dithionite-reduced and dithionite-reduced samples is plotted; (**B**) Bar chart of VHb activity. Data are the means of three independent replicates. Error bars indicate standard deviations; (**C**) Total cell lysates after ultrasonicated treatment. The detailed procedure is described in Section 3.4; (**D**) Visible absorption spectra of crude VHb extracts in the oxygenated (untreated samples) and deoxygenated (dithionite reduced samples) forms.

Subsequently, VHb activity was determined using the CO-difference spectra assay under 25 °C. Samples were reduced with dithionite in the presence and absence of CO. Then the treated samples were scanned on a spectrophotometer. As seen in Figure 3A, the difference spectra of VHb^+^ strain showed a peak in the Soret region at 419 nm, which is characteristic of active VHb. Suddenly, VHb activity obtained at the end of 216 h of 23 °C cultures was 17.8 nmol/g-DCW, which was approximately twofold higher than that obtained at 30 °C (Figure 3B). The finding was in favorable agreement with the fact that the color of total cell lysates of VHb^+^ strain cultured at 23 °C was redder than itself at 30 °C (Figure 3C). This implies that at 30 °C probably there was a significant fraction of VHb that lacked the essential cofactor of heme group. As a result, lower temperature cultures cannot improve VHb expression but can significantly increase VHb activity.

In addition, the VHb expressed at both temperatures exhibited a spectrum characteristic of the oxygenated form. The phenomenon was also observed previously in *Vitreoscilla* [13] and *E. coli* [14] that the expressed VHb existed in the physiologically active ferrous oxygenated form. In our study, the spectrum of the oxygenated VHb obtained from two temperature cultivations had a Soret peak at 415 nm and α and β bands at 580 and 550 nm, respectively (Figure 3D), which represented the hexacoordinated ferrous heme state. After reduced with dithionite (the deoxygenated form) the Soret peak shifted to 425 nm and one broad peak at 562 nm replaced the α and β bands, indicating the pentacoordinated ferrous heme state. The heights of α and β peaks obtained from 23 °C cultivation were more intense than that at 30 °C.

**Figure 4:**
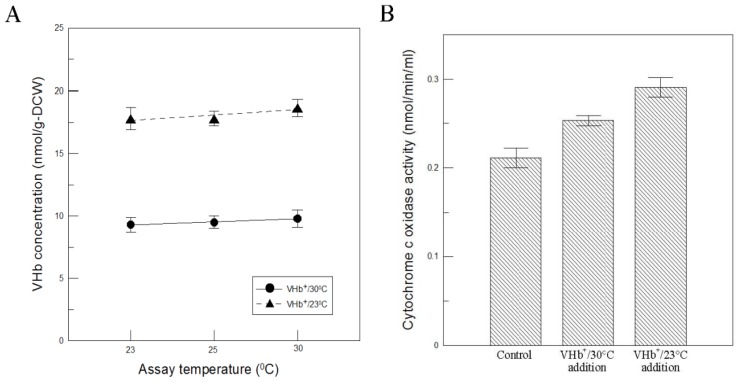
(**A**) The effect of assay temperature on VHb activity. (**B**) The effect of VHb addition on cytochrome c oxidase activity. The cleared lysates of freshly disrupted VHb^−^ cells was used as a control. Data are the means of three independent replicates. Error bars indicate standard deviations.

To clarify if the above results could be caused by the VHb reactivity being more favorable only at lower temperatures, VHb activity was determined at different reaction temperatures. The results shown in Figure 4A indicated that VHb activity obtained at two temperatures cultures did not obviously change within the assay temperatures between 30 °C and 23 °C, thus this reason can be ruled out. Additionally, the exogenous provision of VHb to the assay conditions of the oxygen-consuming enzyme (cytochrome c oxidase) was also carried out to examine if VHb can increase the oxidase activity. First, the cleared lysates of VHb^+^ cells were incubated at 42 °C for 6 h to inactivate cytochrome c oxidase activity. Since cytochrome c oxidase is sensitive to temperatures, whereas VHb can endure temperatures of up to 80 °C [15]. The VHb-containing samples were then added to the cleared lysates of freshly disrupted VHb^−^ cells to measure cytochrome c oxidase activity. As shown in Figure 4B, VHb-containing samples obtained at 23 °C cultures increased cytochrome c oxidase activity by 0.08 nmol min^−1^ mL^−1^, which was twice that obtained at 30 °C cultures. The result was consistent with that shown in Figure 3 and confirmed again that VHb activity obtained at 23 °C cultures was twofold higher than that at 30 °C cultures. It has been proven that VHb can improve respiratory efficiency and ATP synthesis in recombinant hosts [1]. Due to twofold VHb activity achieved, it is reasonable to obtain more biomass and higher viability by VHb^+^ strain at 23 °C than at 30 °C.

**Figure 5:**
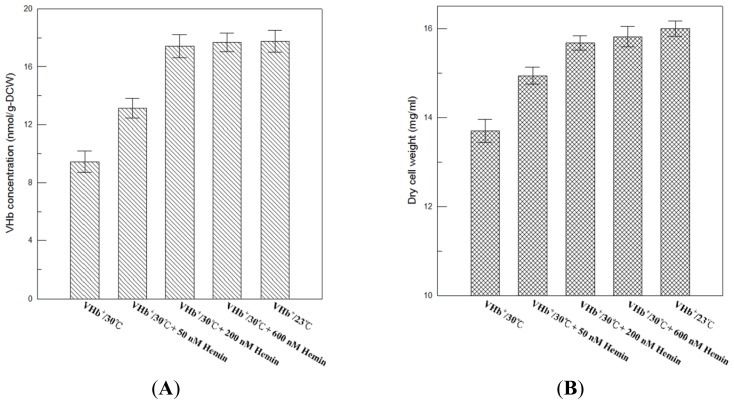
The effects of exogenous provision of hemin on VHb activity (**A**) and cell concentration (**B**) of VHb^+^ strain at the end of 216 h of 30 °C cultures. Data are the means of three independent replicates. Error bars indicate standard deviations.

Our results showed that VHb expression level at 23 °C was equal to that at 30 °C, but VHb activity obtained at 23 °C was twofold higher than that at 30 °C. A similar phenomenon has also been reported in *E. coli* where total globin protein synthesis was barely affected by reducing the temperature from 30 °C to 26 °C, whereas lower temperature cultures produced a twofold higher soluble rHb1.1 than higher temperature cultures did [16]. A significant difference between active VHb at 23 °C and 30 °C could be caused by improper VHb folding that lower temperature cultures can reduce protein synthesis rate, improve protein folding, and increase protein solubility, or due to inadequate heme incorporation that lower temperature cultures could favor heme biosynthesis so that improve heme incorporation, folding, and subunit assembly. In order to determine exactly why in the difference, the exogenous provision of hemin to 30 °C culture broth of VHb^+^ strain was performed to examine if the availability of heme is lower at 30 °C in *P. pastoris*. As shown in Figure 5A, VHb activity of VHb^+^ strain at the end of 216 h of 30 °C cultures increased with hemin concentration and saturated at 200 nM hemin supplement, which was the same level as that of 23 °C cultures (17.8 nmol/g-DCW). This reveals that the amount of heme in *P. pastoris* at 30 °C was lower than that at 23 °C, resulting in lower VHb activity at 30 °C than 23 °C. Therefore, a different heme production in *P. pastoris* caused this twofold difference between active VHb at 23 °C and 30 °C. In addition, a similar effect of hemin on final cell concentration was observed (Figure 5B). Increase of VHb activity caused by the exogenous provision of hemin also improved final cell concentration, and the final biomass of 30 °C culture supplemented with 200 nM hemin was close to that of 23 °C culture.

Lower temperature cultures can increase the availability of oxygen and ATP in *P. pastoris*. Evidences of higher intracellular ATP levels and increased the ATP regeneration rates [17] were obtained at lower temperature cultures. The saturated dissolved oxygen concentration of YPD medium at 23 °C is 7.12 ppm, which is 17% higher than that at 30 °C (6.08 ppm). Increased amounts of oxygen and ATP could affect the flux of several important biosynthetic routes in *P. pastoris*. Increased oxygen concentration can improve the respiration rate and thereby enhanced the oxygen uptake rates (Figure 7). Oxygen tension also influences the folding and assembly of hemeproteins by affecting the availability or redox state of heme. In addition, oxygen is required for two steps in heme biosynthesis, the formation of protoporphyrinogen IX by coproporphyrinogen III oxidase, and the formation of protoporphyrin IX by protoporphyrinogen IX oxidase. The step catalyzed by coproporphyrinogen III oxidase is rate limiting [18]. VHb is a homodimeric protein, and each protomer incorporates a heme prosthetic group in its native state. VHb requires the presence of adequate heme production during VHb protein biosynthesis and folding. Higher amounts of heme could be produced at 23 °C than at 30 °C because lower temperature cultures can increase the availability of dissolved oxygen and ATP, which are used in several steps of heme biosynthesis [18]. Higher availability of heme could favor heme incorporation, folding, and subunit assembly of VHb protein. As a result, more holoVHb (VHb protein carrying heme) could be obtained at 23 °C and resulted in twofold higher VHb activity than that at 30 °C. However, more apoVHb (VHb protein lacking heme) could be produced at 30 °C due to lower availability of heme. The statement also can be confirmed by the result of Figure 3C that more VHb missing heme at 30 °C resulted in a great difference in red color compared to that of 23 °C.

### 2.4. Effect of VHb Expression on Recombinant β-Galactosidase Production at Lower Temperatures

**Figure 6:**
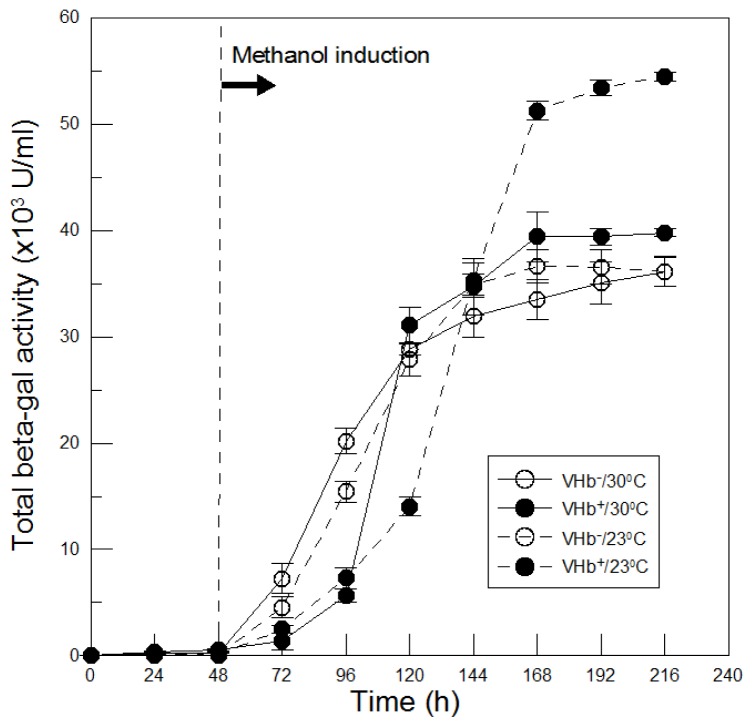
Time courses of total β-galactosidase activity by VHb^+^ and VHb^−^ strains cultured at two temperatures. Data are the means of three independent replicates. Error bars indicate standard deviations.

The differences in recombinant β-galactosidase production between VHb^+^ and VHb^−^ strains at two temperatures were compared. As shown in Figure 6, before induction no β-galactosidase activity was detected in both strains at two temperatures. However, after the absence of glucose (Figure 1B,C) and thereby methanol induction at 48 h of cultures, β-galactosidase began to express. At 30 °C β-galactosidase of VHb^−^ strain was produced immediately and increased with time, leading to a maximal value of 36,000 U/mL achieved at the end of 216 h of cultures. While β-galactosidase of VHb^+^ strain differed from that of VHb^−^ strain. In the early methanol induction phase, β-galactosidase production of VHb^+^ strain was slower than that of VHb^−^ strain. This could be since recombinant VHb protein was co-induced by methanol in VHb^+^ strain; VHb consumed some of cellular resources that could be used for β-galactosidase biosynthesis. However, expressed VHb protein could compensate VHb^+^ strain for improving β-galactosidase production. Therefore, after 120 h of cultures β-galactosidase production of VHb^+^ strain exceeded that of VHb^−^ strain and then reached a maximal activity of 40,000 U/mL at the end of cultures, which was enhanced 11% by VHb effect.

β-Galactosidase production of VHb^−^ strain at 23 °C was analogous to itself at 30 °C and their maximal activities were nearly equivalent. This indicates that β-galactosidase produced by VHb^−^ strain was independent of culture temperatures, which differed from some previous studies reporting that lower temperature cultures can improve target protein production in *P. pastoris* [10–12]. Therefore, the effect of lower temperature cultures on recombinant protein production depends on different target proteins. Comparing β-galactosidase produced at 23 °C by two strains, β-galactosidase production of VHb^+^ strain was also slower in the early methanol induction phase due to co-induced two proteins as mentioned above. Later, however, VHb effect significantly accelerated β-galactosidase production and eventually reached a maximal activity of 55,000 U/mL, which was 50% much higher than that of VHb^−^ strain. Additionally, after 144 h of cultures β-galactosidase production of VHb^+^ strain at 23 °C surpassed itself at 30 °C, and finally obtained an increase of 38% at the end of cultures. This enhancement production of β-galactosidase is not due to lower temperature cultures improving β-galactosidase production but caused by twofold VHb activity obtained at 23 °C cultures.

The results demonstrated that 23 °C cultures further increased VHb activity as to enhance VHb effect on β-galactosidase production as well as on biomass. For various proteins expression in different hosts, culture temperatures cannot usually satisfy both the biomass and protein expression simultaneously. But it can be achieved in this study by culturing *P. pastoris* at 23 °C and obtained a significant higher biomass and β-galactosidase production than that at 30 °C, primarily due to VHb function.

### 2.5. Effect of VHb Expression on Oxygen Uptake Rates at Lower Temperatures

**Figure 7:**
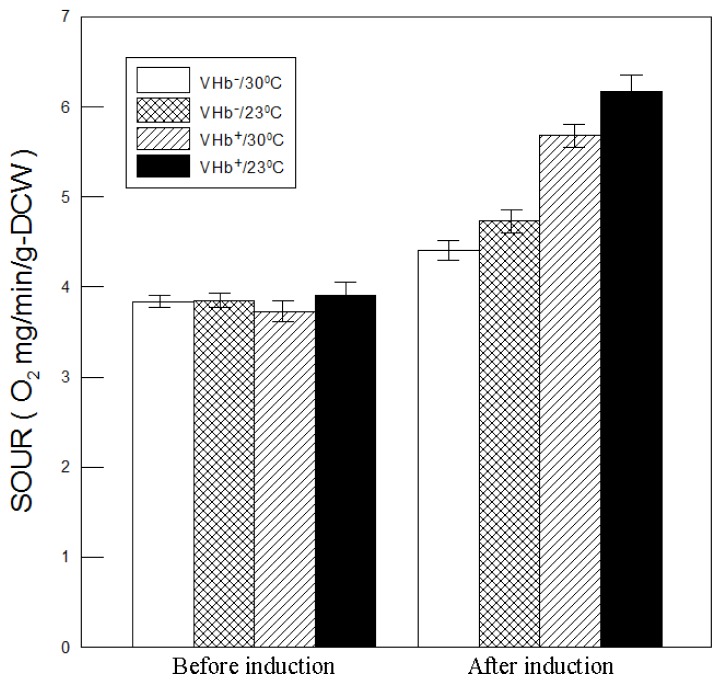
Comparison of specific oxygen uptake rates (SOURs) of VHb^+^ and VHb^−^ strains cultured at two temperatures. The values for before induction were measured after 24 h of culture, and the values for after induction were the maximal values measured after induction. Data are the means of three independent replicates. Error bars indicate standard deviations.

VHb^+^ strain capable of higher biomass and β-galactosidase production implies that it could have a higher oxygen uptake rate. To explore this possibility, the specific oxygen uptake rates (SOURs) were measured. As shown in Figure 7, SOURs of both strains at two temperatures before methanol induction exhibited no obvious differences (around 3.8 O_2_ mg min^−1^ g-DCW^−1^). After induction, however, all SOURs rose to peak values of much higher than 3.8 O_2_ mg min^−1^ g-DCW^−1^ due to an increased oxygen demand from cell growth and methanol metabolization, which is a high oxygen-consuming process. SOURs of VHb^−^ strain cultured at 30 °C and 23 °C increased from 3.8 before induction to 4.4 and 4.7 O_2_ mg min^−1^ g-DCW^−1^ after induction, respectively. While that of VHb^+^ strain rose respectively to 5.6 and 6.3 O_2_ mg min^−1^ g-DCW^−1^. The considerably higher increases in VHb^+^ strain than VHb^−^ strain were probably caused by VHb expression improving oxygen utilization and respiratory efficiency. SOURs of VHb^+^ strain at 30 °C and 23 °C were respectively 27% and 34% higher than that of VHb^−^ strain, indicating that VHb effect on SOURs was beneficial at both temperatures but higher at 23 °C. The VHb-expressing strain with a higher oxygen demand has also been reported previously [19–21].

Moreover, after induction VHb^−^ strain had a higher SOUR at 23 °C than at 30 °C, suggesting that cells at 23 °C demonstrated higher respiratory efficiency and metabolic activity. This is because the oxygen-consuming enzyme AOX1 activity is higher at lower temperatures [12], which thus can reduce the toxicity of methanol to cells and thereby increase cell viability, as shown in Figure 1D. The phenomenon was also observed in VHb^+^ strain but its SOUR increase (0.56 O_2_ mg min^−1^ g-DCW^−1^) was twice as that of VHb^−^ strain (0.25 O_2_ mg min^−1^ g-DCW^−1^), which was likely caused by twofold VHb activity obtained at 23 °C cultures.

Overall, VHb^+^ strain had a higher SOUR at 23 °C than at 30 °C, indicating that cell performance of VHb^+^ strain was better at 23 °C. It can reasonably explain why VHb^+^ strain at 23 °C had a higher final cell density, cell viability, and β-galactosidase production. Consequently, lower temperature cultures can enlarge VHb effect on cell performance of *P. pastoris*.

## 3. Experimental Section

### 3.1. Strains and Culture Conditions

*P. pastoris* strain GS115/Z_A_ (GS115/His^+^Mut^+^/β-gal) was purchased from Invitrogen. The recombinant *P. pastoris* strain GS115/Z_A_SVC (GS115/His^+^Mut^+^/β-gal VHb; VHb^+^ strain) that produces cytosolic β-galactosidase and VHb both under the control of AOX1 promoter was obtained from our previous study [6]. Shake-flask batch cultures were performed at two temperatures of 30 °C and 23 °C to study the effects of VHb expression and lower temperature cultures on cell performance of *P. pastoris*. Recombinant strains were incubated at 250 rpm in 250-mL shake flasks containing 50 mL of YPD medium (1% yeast extract, 2% peptone, 2% dextrose). After glucose as well as ethanol completely exhausted at 48 h of culture, methanol was added to culture medium to a final concentration of 0.5% (*v*/*v*) every 24 h to induce both VHb and β-galactosidase expression. The concentration of glucose and ethanol was determined using HPLC method (Acme 9000 HPLC system, Younglin Instrument Co., Anyang, Korea). Samples were collected at regular intervals to determine protein activity and cell growth, which were monitored by measuring the optical density at 600 nm (OD_600_) using a spectrophotometer (Varian Cary 50 UV/Vis, Walnut Creek, CA, USA). The biomass was also determined gravimetrically by performing a dry cell weight (DCW) measurement.

### 3.2. Measurement of Cell Viability

Cell viability was measured using the methylene blue dye exclusion technique, as previously described by Wang *et al.* [10]. Culture samples were obtained at regular intervals, and a suitably diluted cell suspension was mixed with an equal volume of methylene blue dye solution, containing 0.01% (*w*/*v*) methylene blue and 2% (*w*/*v*) tri-sodium citrate dihydrate in phosphate-buffered saline solution (137 mM NaCl, 2.7 mM KCl, 10 mM Na_2_HPO_4_, 1.8 mM KH_2_PO_4_; pH 7.4). Samples were then mounted on a hemocytometer under an optical microscope (Olympus CX31, Tokyo, Japan), and the percentage of live cells in the total population was calculated. The cells that had absorbed methylene blue and appeared dark blue were considered dead, while those that appeared translucent were regarded as live.

### 3.3. Measurement of Oxygen Uptake Rate

The oxygen uptake rate (OUR) was determined using the dynamic gassing-out method described by Urgun-Demirtas *et al.* [22]. Recombinant *P. pastoris* strains were harvested at regular intervals using centrifugation at 6000*g* for 10 min. Cell pellets were washed twice with the air-saturated YPD medium and then resuspended in 80 mL of the same medium. A magnetic impeller was installed in the bottle of reaction chamber, which was tightly sealed using an oxygen probe (YSI Model 5100 dissolved oxygen meter, Yellow Springs, OH, USA). Time course of the decrease in the dissolved oxygen concentration was recorded, and the slope of dissolved oxygen *versus* time plot was used to determine the OUR. The specific oxygen uptake rate (SOUR), defined as the ratio of OUR to DCW, was expressed as milligrams of oxygen consumed per minute per g-DCW.

### 3.4. Analysis of Protein Expression and Activity

Ortho-nitrophenyl-β-D-galactopyranoside (ONPG, Sigma, St. Louis, MO, USA) was used as the reaction substrate to measure β-galactosidase activity. Recombinant *P. pastoris* cells were first permeabilized with 40% isopropanol [23], and β-galactosidase activity was then assayed using the procedure described in the *Pichia* Expression Kit (Invitrogen, Carlsbad, CA, USA). One enzyme unit is defined as the quantity of enzyme that catalyzes the liberation of 1 μmol of ortho-nitrophenol from ONPG per minute under the assay conditions.

To determine VHb activity, the carbon monoxide (CO)-difference spectra assay of crude protein extract was recorded, as described previously [24]. Cell pellets were harvested using centrifugation at 6000*g* for 10 min and washed twice with the breaking buffer (50 mM sodium phosphate (pH 7.4), 1 mM PMSF, 1 mM EDTA, and 5% glycerol). The pellets were then resuspended in the same buffer and sonicated in an ice bath using an ultrasonifier, followed by centrifugation at 12,000*g* for 15 min to remove cell debris. The supernatants thus obtained were used as the crude protein extracts, which were reduced with sodium dithionite and bubbled with CO to analyze VHb activity. The CO-difference spectra were recorded from 400 to 500 nm using a spectrophotometer (Varian Cary 50 UV/Vis, Walnut Creek, CA, USA). The absorbance of a VHb sample in the sample cuvette that had been bubbled with CO and an otherwise identical sample of VHb that had not been bubbled with CO were recorded, and the difference absorption spectra between CO-dithionite-reduced and dithionite-reduced samples were determined. VHb concentration was calculated using the extinction coefficient E_419–436 nm_ = 274 mM^−1^cm^−1^, as previously described [14].

Cytochrome c oxidase activity was determined using a colorimetric assay based on the oxidation of reduced cytochrome c to ferricytochrome c by cytochrome c oxidase. The reaction can be followed by a decrease in the absorbance at 550 nm under the following conditions. Samples were incubated at 25 °C with 10 mM Tris-HCl, pH 7.0, 250 mM sucrose, 120 mM KCl, and 0.01 mM ferrocytochrome c for 5 min with 10-s intervals [25].

The crude protein extracts were analyzed using SDS-PAGE and stained with Coomassie brilliant blue. Proteins that had been separated on a 12% Tris-glycine gel were transferred to a PVDF membrane (Hybond-P, Amersham Pharmacia, Piscataway, NJ, USA) using electroblotting with a transfer apparatus (Mini Trans-Blot Cell, Bio-Rad, Hercules, CA, USA) for Western- blotting analysis. After blotting, the PVDF membrane was soaked with the blocking buffer (5% *w*/*v* skimmed milk powder in Tris-buffered saline) and treated with the rabbit anti-VHb antibody and mouse anti-β-galactosidase antibody for 2 h. The membrane was then washed three times with TBS, followed by treatment with the goat anti-rabbit and goat anti-mouse secondary antibody for 2 h, both conjugated with alkaline phosphatase. The membrane was again washed three times with TBS, and the image was developed using the Immun-Blot AP kit (Bio-Rad) to detect the expression of VHb and β-galactosidase.

## 4. Conclusions

Lower temperature cultures at 23 °C can enlarge VHb effect on cell performance of *P. pastoris* in order to obtain a higher final cell density, viability, SOUR and β-galactosidase production in comparison with 30 °C cultures, presumably owing to the twofold VHb activity obtained at 23 °C cultures. A greater fraction of VHb inactive at 30 °C, compared with that at 23 °C, was caused by the absence of heme at 30 °C. The strategy of combining heterologous VHb expression and lower temperature cultures makes *P. pastoris* an excellent expression platform particularly suitable for increasing the yields of the aggregation-prone and low-stability recombinant proteins.
